# A comparative study of disease genes and drug targets in the human protein interactome

**DOI:** 10.1186/1471-2105-16-S5-S1

**Published:** 2015-03-18

**Authors:** Jingchun Sun, Kevin Zhu, W Jim Zheng, Hua Xu

**Affiliations:** 1 School of Biomedical Informatics, The University of Texas Health Science Center at Houston, Houston, TX 77030, USA; 2 Graduate School of Biomedical Sciences, The University of Texas Health Science Center at Houston, Houston, TX 77030, USA

## Abstract

**Background:**

Disease genes cause or contribute genetically to the development of the most complex diseases. Drugs are the major approaches to treat the complex disease through interacting with their targets. Thus, drug targets are critical for treatment efficacy. However, the interrelationship between the disease genes and drug targets is not clear.

**Results:**

In this study, we comprehensively compared the network properties of disease genes and drug targets for five major disease categories (cancer, cardiovascular disease, immune system disease, metabolic disease, and nervous system disease). We first collected disease genes from genome-wide association studies (GWAS) for five disease categories and collected their corresponding drugs based on drugs' Anatomical Therapeutic Chemical (ATC) classification. Then, we obtained the drug targets for these five different disease categories. We found that, though the intersections between disease genes and drug targets were small, disease genes were significantly enriched in targets compared to their enrichment in human protein-coding genes. We further compared network properties of the proteins encoded by disease genes and drug targets in human protein-protein interaction networks (interactome). The results showed that the drug targets tended to have higher degree, higher betweenness, and lower clustering coefficient in cancer Furthermore, we observed a clear fraction increase of disease proteins or drug targets in the near neighborhood compared with the randomized genes.

**Conclusions:**

The study presents the first comprehensive comparison of the disease genes and drug targets in the context of interactome. The results provide some foundational network characteristics for further designing computational strategies to predict novel drug targets and drug repurposing.

## Background

In the past decade, discovering the genes underlying disease susceptibility in human (disease genes) has been a major task in biomedical research area. These disease genes cause or contribute genetically to the development of the most complex diseases such as cancer [1], diabetes [2], and schizophrenia [3]. Completely understanding of the genetic predisposition of complex diseases is critical for discovering novel drug targets and developing new treatment strategies [4,5]. For example, some of these disease genes have been successfully employed in clinical application to seek more efficient and less toxic therapies. In breast cancer, the germline *BRCA1/BRCA2 *genotyping is used to determine susceptibility to breast and ovarian cancer[6-8] and the elevated expression level of circulating tumor marker HER-2 is used to determine the treatment of the monoclonal antibody Trastuzumab [9,10]. Though the clinical application of these disease genes has fueled discoveries of disease biomarkers and drug targets, successful cases are still very limited compared to the large number of disease genes. Recently, disease genes have been regarded as a major source for determining novel drug targets for human disorders [11-13]. However, how the drug targets influence the disease genes is not clear. Thus, we hypothesized that investigating their relationship in the context of networks will provide some novel insights for further understanding of the molecular mechanism underlying treatment, which in turn may facilitate the identification of novel drug targets and the development of computational approaches for drug repurposing and drug combinations.

With the development of large-scale technologies such as genome-wide association study (GWAS) and next generation sequencing (NGS), molecular and genetic studies of human diseases have discovered an impressive number of associations between genes and various diseases [14,15]. GWAS especially has been used as a major tool to query common genetic variations across the entire human genome in an unbiased fashion. It allowed for the discovery of a number of new gene regions contributing to multifactorial diseases [16]. More importantly, the National Human Genome Research Institute (NHGRI) Catalog of Published Genome-wide Association Studies (GWAS catalog) has a collection of all published GWAS [17], which provides comprehensive insights into the functional implication for human diseases and traits at the systematic level. Likewise, multiple drug-centered databases such as DrugBank [18] and PharmGKB [19] provide comprehensive data for systematic analysis. The extensive data from both areas makes it possible and practical to investigate the interrelationship between disease genes and drug targets at the systemic level. Although the data may be incomplete or error-prone, it nonetheless offers us an unprecedented opportunity to uncover their connections. Recently, a network-based approach to human disease (network medicine) has become a major and powerful tool for identifying new disease genes, uncovering the biological significance of disease-associated mutations, and identifying drug targets and biomarkers for complex diseases [15,20-23]. In biological networks, the implications of the topological properties of the nodes can provide an overview of the organizing principles that govern both the networks and nodes' biological meanings [24-26]. Therefore, our goal here is to understand the interrelationship between disease genes and drug targets in the context of the human protein-protein interaction (PPI) networks.

In this study, we determined five different disease categories based on the disease and traits from the GWAS data and then collected the disease genes and drug targets for the common disease. In the context of human diseases, drug target genes were defined by mapping the drug targets to their encoding genes, while, in the context of the PPI network, the nodes mapping to disease genes were defined as disease proteins. We first compared the overlap between the disease genes and drug target genes. Then, by mapping the disease genes and drug target genes onto the human interactome, we calculated the three basic topological measurements, i.e., degree, betweenness, and clustering coefficient. We further examined the neighborhoods of disease proteins by quantifying the fraction of drug targets that were in an eighth-degree neighborhood around a disease protein. The same was done in the neighborhoods of drug targets and the fraction of disease proteins. This study first systematically investigated the interrelationship between the disease genes and drug targets in the context of networks, providing some foundational network characteristics for further design of computational strategies for predicting novel drug targets and drug repurposing.

## Materials and methods

### Disease genes from GWAS catalog

In this study, the disease genes were defined as the reported genes provided by the NHGRI Catalog of Published GWAS Catalog [27]. The GWAS catalog provides a quality-controlled, manually-curated, literature-derived collection of all published GWAS [17]. It collects the published information such as PubMed ID, reported genes, SNP-located genes, SNPs, and SNP-trait association *P*-values from the GWAS and has assayed at least 100,000 SNPs, listing those SNP-trait associations with *P*-values less than 1.0 × 10^-5^. We downloaded the data from the GWAS catalog website https://www.genome.gov/26525384, November 2013). Additionally, the European Bioinformatics Institute (EBI) Functional Genomics Production Team generated a GWAS diagram browser using semantic web technologies http://www.ebi.ac.uk/fgpt/gwas/ for exploring the GWAS [27]. Along with generating the browser, the researchers have formalized the GWAS traits by mapping each trait to one or more terms through the Experimental Factor Ontology (EFO). We downloaded the EFO mapping file (November 2013), which includes the relationship between each study trait to the thirteen parent SNP-associated trait categories. Among the thirteen trait categories, six were directly related to one major type of disease, such as cancer, cardiovascular disease, digestive system disease, immune system disease, metabolic disease, and nervous system disease. Considering that digestive diseases (gastrointestinal disorders) belong to code A (Alimentary tract and metabolism) in the drug Anatomical Therapeutic Chemical (ATC) classification system, we grouped the digestive and metabolic diseases into the same category. Thus, in this study, there were five different disease categories: cancer, cardiovascular disease, immune system disease, metabolic disease, and nervous system disease. For each disease category, genes with SNPs having GWAS significant *P*-values of less than 1.0 × 10^-8 ^were defined as disease genes [28]. Then, we matched them to the gene IDs and gene symbols from the latest version of gene annotation downloaded from the NCBI human reference genome Entrez Gene [29] to represent the disease genes.

### Drugs and their targets

We downloaded the DrugBank database (version 3.0) as of October 2013. DrugBank combines comprehensive drug-related information data including the drug-target and drug ATC code information [18]. For each drug, we extracted several fields such as "Name", "Drug Target", and "ATC Codes" data to obtain primary drug targets and drug ATC annotations. We utilized the DrugBank drug IDs and drug names to represent the drugs. For drug targets, we first downloaded the unique UniProtKB accession numbers (ACs) to represent protein targets and matched them to gene symbols through two steps. First, we mapped UniProtKB ACs to Entrez gene IDs by the ID Mapping tool in the UniProt database [30] and then mapped gene IDs to gene symbols according to the latest annotation file used in the disease gene mapping.

### Matching between GWAS traits and drug indications using ATC classification

To match drug indications to GWAS traits, we utilized the drug Anatomical Therapeutic Chemical (ATC) classification http://www.whocc.no/atc_ddd_index/. The classification system groups the active drugs into five different levels based on the organ or system on which they act as well as their therapeutic and chemical characteristics. Its first level has fourteen anatomical main groups, of which each is represented by one letter. For example, N represents "nervous system." The second sublevel of the ATC coding system contains systems-specific therapeutic subgroups represented by a two-digit number. For example, N05 represents "psycholeptics," a therapeutic subgroup of the anatomical group N "nervous system."

To complement the drug ATC annotation from the DrugBank database, we further utilized ATC classification annotations for each drug from the Kyoto Encyclopedia of Genes and Genomes (KEGG) drug database [30]. We downloaded the drug information file from KEGG FTP website ftp://ftp.genome.jp/pub/kegg/medicus/ in November 2013 and then extracted the ATC annotation for each drug. We then merged the ATC annotation from the two databases by removing redundancies.

We manually searched the ATC codes for five different disease categories and then matched the drug indications to disease categories through the following drug ATC codes. The drugs belonging to the L01 (Antineoplastic agents) class were defined as treatment of cancer; the drugs belonging to the L03 (Immunostimulants) and L04 (Immunosuppressants) as the treatment of immune system diseases; the drugs belonging to the N (Nervous system) as the treatment of nervous system disease; the drugs belonging to the C (Cardiovascular system) as the treatment of cardiovascular system diseases; and the drugs belonging to the A02 (Drugs for acid related disorders), A03 (Drugs for functional gastrointestinal disorders), A04 (Antiemetics and antinauseants), A05 (Bile and liver therapy), A06 (Drugs for constipation), A07 (Antidiarrheals, intestinal anti-inflammatory/anti-infective agents), and A10 (Drugs used in diabetes) as the treatment of metabolic diseases. For those that mapped to different categories of diseases, we assigned them to multiple disease groups.

### Protein-protein interaction data and analysis

We downloaded the latest version of the human PPI data from the Protein Interaction Network Analysis platform (PINA v2.0) (http://csbi.ltdk.helsinki.fi/pina/, September 2013) [31]. We retrieved the experimentally verified PPI data for further analysis and then mapped the protein identifications to the official gene symbols. After removing redundancies and self-interactions, we obtained one human PPI network, which included 101,219 edges connecting 12,978 nodes.

In this study, we calculated the three basic network topological measures, i.e., degree, betweenness, and clustering coefficient, in order to examine network properties of ten sets of proteins in the PPI network, as described in our previous study [25]. In the biological networks, the degree of a node (connectivity) is the count of its direct links, which is the most basic network property., The more links a node has, the more important it is in terms of network stability [32]. Second, the betweenness of a node is defined as the number of shortest paths between all possible pairs of nodes in the network that traverse the node. It measures the ways in which signals can pass through the interaction network [33]. Finally, the clustering coefficient of a node is the ratio of the observed number of direct connections between the node's immediate network neighbors over the maximum possible number of such connections. It measures the density of its neighborhood, which means weather or not the node's interactors could form modules. In biological networks, the components in a module often work together to achieve a relatively distinct function.

To further investigate the interrelationship between disease proteins and drug targets in the human interactome, we examined their neighborhoods by quantifying the fraction of drug targets in the disease protein neighborhood or the fraction of disease proteins in the drug target neighborhood. To do this, we examined the eight-degree level of the neighborhood for each node. For example, for a given disease protein A, we collected the nodes that have direct links with it as its first-degree neighbors. From its first-degree neighbors, we collected the nodes having direct links with them and excluded the disease proteins as its second-degree neighbors. From its second-degree neighbors, we collected the nodes that have direct links with second-degree neighbors and excluded its first-degree neighbors as its third-degree neighbors. We continued this pattern five more times, ending on the eighth-degree neighbors. For a given disease protein at a given degree, we calculated the fraction of targets in its neighbors as the number of nodes belonging to targets divided by the number of targets. For a given target, For a given target at a given degree, we calculated the fraction of disease proteins in its neighbors as the number of nodes belonging to disease proteins divided by the number of disease proteins.

## Results and discussion

### Disease genes and drug target genes

**Figure 1:**
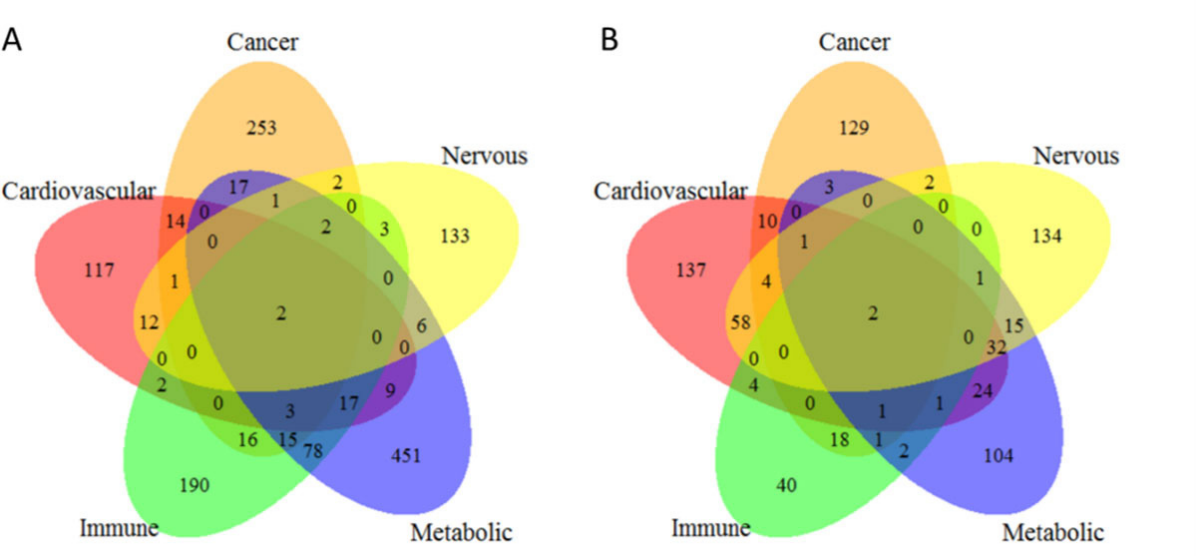
**Comparisons among five sets of disease genes (A) and five sets of drug target genes (B) for five disease categories**. The five disease categories included cancer, cardiovascular disease, immune system disease, metabolic disease, and nervous system disease.

Using the GWAS association results from the GWAS catalog, we obtained five sets of disease genes with SNPs having significant *P*-values of less than 1.0 × 10^-8^. Then, based on the GWAS trait classification from EBI, we classified the gene sets into five different disease categories (cancer, cardiovascular disease, immune system disease, metabolic disease, and nervous system disease) for further analyses. Thus, we obtained a total of 1,344 disease genes from 450 GWA studies of five disease categories (Table 1). The numbers for the five sets of disease genes ranged from 162 for nervous disease to 601 for metabolic disease. Figure 1A shows their intersections. Among them, the metabolic and immune disease-associated genes had many common genes (117, 35.67%), which indicated that more than one third of the immune disease genes were reported to be significantly associated with the metabolic disease. This observation was consistent with the idea that the metabolic and immune systems have many links on multiple levels in the biological processes [34].

**Table 1:** Summary of GWA studies, disease genes, SNPs, drugs, and target genes for five disease categories

Disease category	GWAS	Gene	SNP	Drug	Target	Overlap^a^	*P*-value^b^	ATC code^c^
Cancer	147	326	341	115	171	7	0.01	L01
Cardiovascular	52	177	202	230	273	5	0.06	C
Immune	92	328	398	49	70	8	1.3 × 10^-5^	L03, L04
Metabolic	90	601	561	146	185	13	2.0 × 10^-3^	A02, A03, A04, A05, A06, A07, A10
Nervous	74	162	178	314	248	3	0.18	N
Total	450	1,344	1,586	827	719	95	1.6 × 10^-11^	

^a^The number of common genes between disease genes and drug target genes for each disease category.

^b^The *P*-value was calculated by hypergeometric test on the common genes between disease genes and target genes compared with the human protein-coding genes for each disease category. ^c^The detail information for each ATC code was included in the Materials and methods.

We extracted drugs and their targets from DrugBank database. To match drug indications to GWAS trait classes, we employed the drug ATC classification. We obtained drugs' ATC codes from DrugBank and KEGG databases. Accordingly, we obtained a total of 827 drugs for five disease categories (Table 1). Among them, 25 drugs belonged to two categories of disease and one drug belonged to three categories. Thus, we classified a total of 719 targets into five different disease categories. The numbers of the five sets of drug target genes ranged from 70 for immune disease to 273 for nervous disorder. Figure 1B shows their intersections. Among these overlapped drug target genes, one-third of the target genes for cardiovascular disease also belonged to the target genes for nervous disease (97, 35.53%) and one-fourth (25.74%) belonged to the target genes for metabolic disease. These observations further confirmed that both the nervous system and metabolic syndrome play important roles in the regulation of cardiovascular function over multiple time scales [35,36].

Table 1 summarizes the disease genes, drugs, drug target genes, and the ATC codes of drugs for matching drug indications to GWAS traits. We further examined the overlaps between the disease genes and drug target genes for each disease category. The number of genes common to disease genes and drug target genes was very small for all five types of diseases (Table 1). However, when compared to human protein-coding genes, the disease genes tended to be enriched in the known drug target genes. Overall, there were 95 common genes (5.07%) between 1,344 disease genes and 719 drug target genes. To further evaluate the significance of enrichment for disease gene set in target gene set, we performed the hypergeometric test (PubMed ID: 15980575) compared with 719 drug target genes of 20,629 human protein-coding genes. The result indicated that the overall disease gene set was enriched in the target gene set, which was more than expected by chance (Hypergeometric test *P*-value: 1.6 × 10^-11^). The observation indicated that the disease genes tend to be druggable, which is consistent with the previous results that GWAS genes are significantly more likely to be theoretically druggable or biopharmable targets than expected by chance [12]. Similarly, we further assess the significance of the enrichment for disease gene set in target gene set for each disease categories. Compared to the 791 drug target genes of 20,629 human protein-coding genes, the disease gene set was significantly enriched in target genes in cancer, immune disease, and metabolic disease (*P*-values: 0.01, 1.3 × 10^-5^, and 2.0 × 10^-3^, respectively) but not in the cardiovascular and nervous diseases (*P*-values: 0.06 and 0.18, respectively) (*P*-value < 0.05). This inconsistency might reflect that different disease categories have different molecular mechanisms, or it might be due to the study bias of the different disease categories in biomedical research [37]. More effort could be made for further improvement of the disease gene data by including both more general disease databases, such as the Online Mendielian Inheritance in Man (OMIM) [38] and the Genetic Association Database (GAD) [39], and specific disease gene databases, such as Catalogue Of Somatic Mutations in Cancer (COSMIC) [40] for cancer and SchizophreniaGene [41].

### Network properties of disease proteins and drug targets in the human interactome

**Figure 2:**
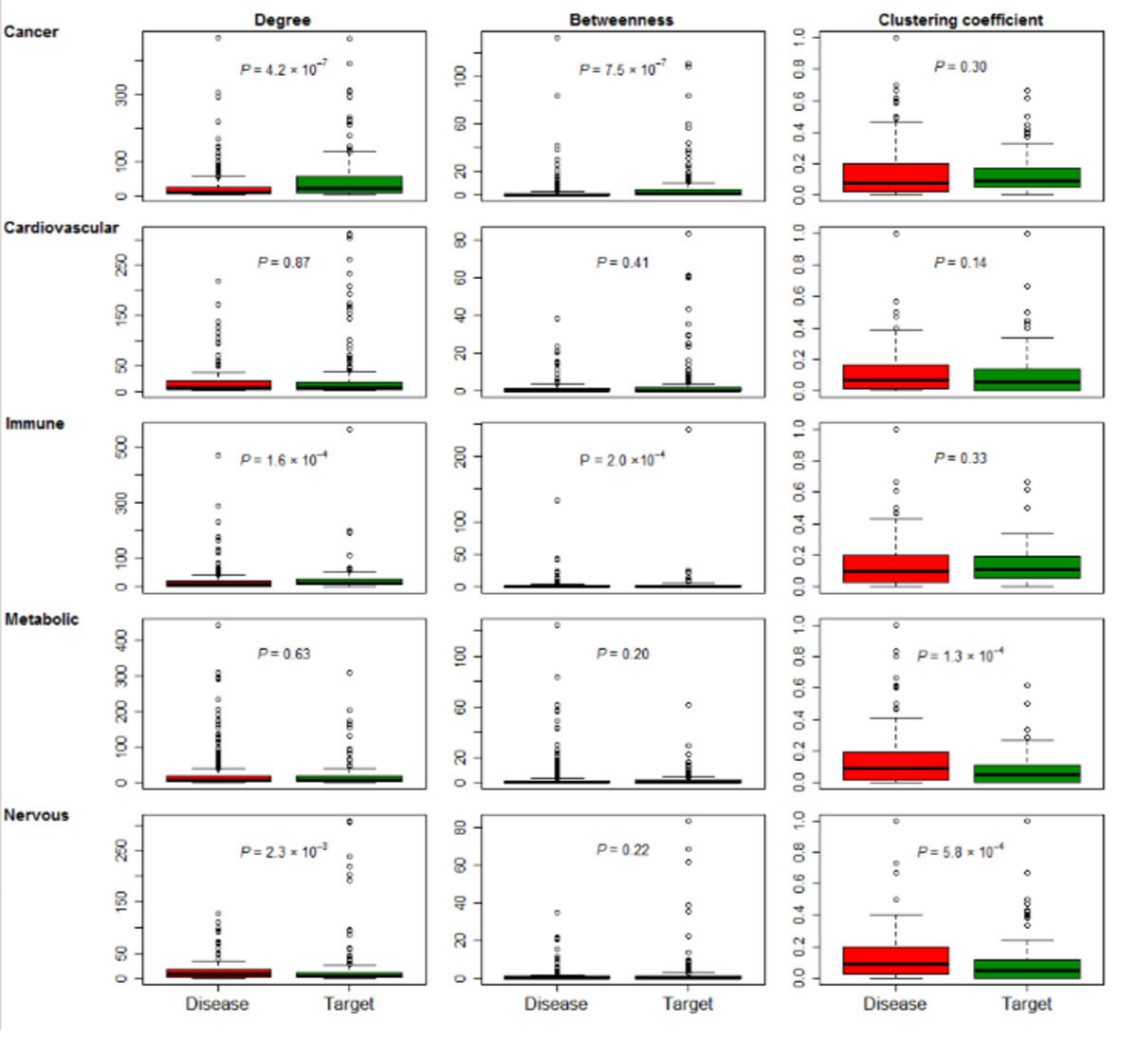
**Comparison of three network properties of disease proteins and drug targets in the five disease categories**.

For each node in the network, the degree, betweenness, and clustering coefficient, are mostly basic network properties. In this study, we examined and compared the three network measurements of five pairs of the disease gene proteins and drug targets for five disease categories (cancer, cardiovascular disease, immune disease, metabolic disease, and nervous disease). Table 2 summarizes the network properties of the five sets of disease proteins and five sets of drug targets and Figure 2 shows the comparison of the distributions of five network properties of disease proteins and drug targets in the human interactome.

**Table 2:** Summary of network properties for disease genes and drug target genes in the human interactome

Disease category	No. of proteins		Degree		Betweenness (1.0 × 10^4^)		Clustering coefficient	
				
	Disease	Target	Disease	Target	Disease	Target	Disease	Target
Cancer	270	171	24.59	48.34	2.78	6.40	0.14	0.13
Cardiovascular	145	273	19.43	25.41	2.04	3.56	0.13	0.11
Immune	264	70	20.47	32.41	2.19	5.69	0.16	0.14
Metabolic	487	187	21.13	18.56	2.51	2.91	0.15	0.08
Nervous	125	249	16.68	23.25	1.81	2.73	0.17	0.11
Total	1065	643	20.25	26.12	2.23	3.51	0.15	0.12

The node's degree is the most elementary characteristic of a node, which allows us to compare and characterize different gene sets. Overall, the average degree of drug targets tends to be higher than that of the disease proteins in the same disease category. Among the five disease categories, the average degrees of three drug targets in three disease categories (cancer, immune, or nervous) were significantly higher than that of the corresponding disease proteins (Wilcoxon test, *P *= 4.2 × 10^-7^, 1.6 × 10^-4^, 2.3 × 10^-3^, respectively). Interestingly, the average degree of the cancer drug targets was approximately two times that of the cancer disease proteins. This shows that cancer drug targets tend to interact strongly with other proteins and have higher degree in the whole network. This observation might reflect that the cancer disease genes and cancer target genes mainly play roles in different biological processes [12]. To elucidate this difference, we would need to integrate more data from other cancer-related gene sources such as cancer gene census [37] and genes with somatic recurrent mutations from The Cancer Genome Atlas (TCGA) project [42].

For each node, the betweenness measures the number of shortest paths between all pairs of nodes in the network that pass through the node, which may reflect the extent of signals that might have paths through the node in a biological network. Similarly, for the five different disease categories, the overall average betweenness of the drug targets was higher than of the disease genes. For example, the average betweenness of the cancer drug targets was significantly greater than that of the cancer disease proteins (Wilcoxon test, *P *= 7.5 × 10^-7^). A similar difference could be seen between the immune drug targets (*P *= 7.5 × 10^-7^) and the immune disease proteins (*P *= 2.0 × 10^-3^), while for cardiovascular, metabolic, nervous diseases, the comparison did not reach significance. Finally, a higher clustering coefficient of a node indicates a higher density of its network connections. The average clustering coefficient of the target proteins in cancer, cardiovascular disease, and immune disease were not significantly lower than the corresponding average clustering coefficient in each category (*P *= 0.30, 0.14, and 0.33, respectively) but the average clustering coefficient of the target proteins in metabolic and nervous disease categories were significantly lower than the corresponding average clustering coefficient in each category (*P *= 1.3 × 10^-4 ^and 5.8 × 10^-4^, respectively).

Overall, we found that disease drug targets had higher degree, higher betweenness, and lower clustering coefficients, some of which even reached statistical significance in certain disease categories. These observations not only indicated that drug targets had different network properties compared to disease proteins but also suggested that drugs perform their actions through their targets disrupting the disease-related modules. Therefore, when applying network properties to predict drug targets and potential drug repurposing, it might be necessary to perform a comprehensive investigation of those network properties.

### Neighborhood of disease proteins and drug targets in human interactome

**Figure 3:**
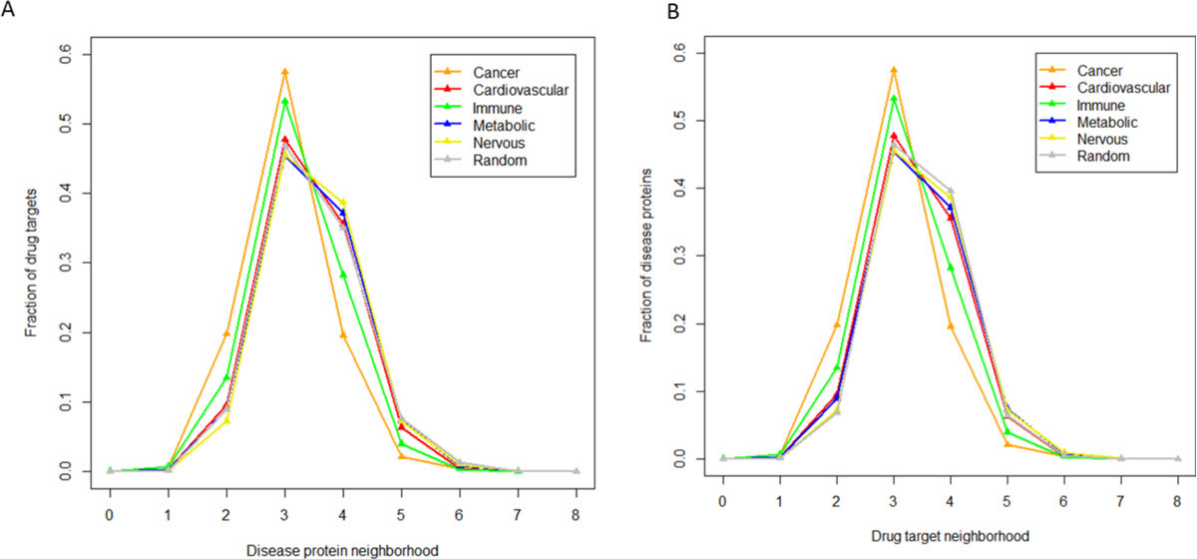
**Neighborhood of disease proteins (A) and drug targets (B) in five different disease categories**.

Figure 3A shows the fraction of drug targets in the disease protein neighborhood while Figure 3B shows the fraction of disease proteins in the drug target neighborhood. The results show that, for all disease genes and the drug targets in all diseases, there was an increased enrichment in the region of the first-degree, second-degree, and third-degree when compared with the randomized nodes to the total disease gene size or the drug target size, which is consistent with the results observed by Yildirm et al. using the shortest path distance to compare the disease proteins from the OMIM and drug targets [43].

To further examine if the fraction of disease genes in the target neighborhoods, or the fraction of targets in the disease gene neighborhood, is higher than that of randomized nodes, we performed the one-sided Wilcoxon tests at first-degree, second-degree, and third-degree, respectively. Table 3 summarizes the Wilcoxon test *P*-values. The results showed that, compared to the randomized nodes, the fractions of targets in the disease gene neighborhood or the fractions of disease genes in the target neighborhood are significantly higher than that in the randomized node's neighborhood in the cancer, cardiovascular and immune disorders, respectively (*P*-value < 0.05). However, in the metabolic and nervous disorders, we did not observe the significance. These observations suggest that, different types of disorders have their characteristics, which might be caused by their particular pathology. When applying the relationship between the disease proteins and known drug targets to predict the novel drug targets in the context of the networks, it is necessary to extensively investigate the relationship between the disease genes and drug targets. Even though, our results overall suggest, in cancer, cardiovascular and immune disorder, it might be an appropriate option to utilize the disease gene and target relationship up to third-degree in the context of networks when applying the network properties for drug discovering design.

**Table 3:** Summary of one-sided Wilcoxon tests' *P*-values comparing the disease/target fraction in the target/disease neighbor with those of the randomized nodes at first-degree, second-degree, and third degree

Degree	First-degree	Second-degree	Third-degree
***Disease neighborhood***			
Cancer	2.37 × 10^-6^	7.68 × 10^-15^	2.94 × 10^-9^
Cardiovascular	4.67 × 10^-4^	1.56 × 10^-41^	2.33 × 10^-54^
Immune	0.04	1.28 × 10^-4^	8.12 × 10^-5^
Metabolic	0.72	0.08	0.99
Nervous	0.70	0.46	0.92
***Target neighborhood***			
Cancer	2.82 × 10^-14^	4.67 × 10^-20^	4.76 × 10^-11^
Cardiovascular	0.01	2.24 × 10^-43^	3.62 × 10^-70^
Immune	1.48 × 10^-13^	1.13 × 10^-7^	0.01
Metabolic	5.00 × 10^-5^	0.08	0.60
Nervous	0.84	0.71	0.35

## Conclusion

This study is important because of the slowness of drug discovery and increase of drug discovery cost. The emerging area of computational network pharmacology provides complementary approaches for facilitating the drug development. However, more work is necessary to identify common patterns of drug targets, disease genes, and their interrelationship in the context of networks. The findings in this study could be used to design the computational methods for drug target identification, drug repurposing, and drug combination when taking drug target network as one factor.

## Competing interests

The authors declare that they have no competing interests.

## Authors' contributions

JS participated in the method development, prepared the data, carried out the data analysis, and contributed to the writing of the manuscript. KZ participated in the method development and data analysis. WZ contributed to the writing of the manuscript. HX participated in the method development, and contributed to the writing of the manuscript. All authors read and approved the final manuscript.

## Declaration

The publication funding came from Cancer Prevention & Research Institute of Texas (CPRIT R1307).

This article has been published as part of *BMC Bioinformatics *Volume 16 Supplement 5, 2015: Selected articles from the 10th International Symposium on Bioinformatics Research and Applications (ISBRA-14): Bioinformatics. The full contents of the supplement are available online at http://www.biomedcentral.com/bmcbioinformatics/supplements/16/S5.
